# Supplementary material for: WordCommentsAnalyzer: A windows software tool for qualitative research
Source: F1000Res. 2018 Sep 4;7:536. Originally published 2018 May 3. [Version 2] doi: 10.12688/f1000research.14819.2 (PMC6137411; doi:10.12688/f1000research.14819.2)
Supplement: Supplementary file 3 [file f1000research-7-17614-s0002.tgz › b2a79496-4ada-47c4-9682-a99ce867836d.zip!/Eight Iranian Health Journals/Yafteh _64.docx]

© Yafteh  . This work is licensed under CC BY 4.0 (https://creativecommons.org/licenses/by/4.0/).

Volume & Issue: دوره 5 - شماره 1

Title: اثر عصاره برگ شاه توت، دانه شنبلیله و برگ زیتون بر تغییرات سطح پلاسمایی کلسترول و رگه های چربی در جدار شریان آئورت خرگوش

http://yafte.lums.ac.ir/article-1-955-fa.html

مقدمه : اختلال چربیهای خون یکی از مهمترین فاکتورهای خطر ناک در ایجاد آترواسکلروز شریانهای کرونر قلب هستند. هدف از انجام این مطالعه بررسی اثر عصاره دی اتیل اتری برگ زیتون و برگ شاه توت و دانه شنبلیله بر سطوح لیپیدهای پلاسما و ایجاد پلاکهای آترواسکلروزی بوده است. مواد و روشها : در این مطالعه آزمایشی 45 خرگوش نر سفید در دمای Ċ1± 22 نگهداری و به 9 گروه , پنج تایی تقسیم شدند . به گروه 1و2 به ترتیب رژیم عادی و پر کلسترول و به گروههای 3 تا 9 رژیم پر کلسترول به همراه عصاره های دارویی(بصورت تکی وترکیبی) به مدت 60 روز داده شد. قبل و بعد از مطالعه سطح پلاسمایی لیپیدهاو بعد از مطالعه فشار خون مستقیم حیوانات اندازه گیری شد. همچنین جدار شریان آئورت از لحاظ وجود یا عدم وجود پلاکهای آترواسکلروزی بررسی شد. یافته ها : نتایج نشان داد عصاره دی اتیل اتری برگ زیتون و برگ شاه توت هر کدام به تنهایی سطح پلاسمایی کلسترول تام و LDL را بطور معنی داری کاهش داده اند ؛ اما اثر عصاره دانه شنبلیله بی تاثیر بوده است. از طرفی مصرف عصاره دانه شنبلیله به همراه عصاره برگ شاه توت و برگ زیتون اثر آنها را روی کلسترول تام و LDL تقویت کرد. عصاره برگ زیتون سطح پلاسماییHDL را بالا برد و تری گلیسرید را کاهش داد؛ اما عصاره برگ شاه توت و دانه شنبلیله هیچ گونه اثری بر HDL نداشتند. عصاره برگ زیتون و برگ شاه توت از تشکیل سریع پلاکهای آترواسکلروزی جلوگیری نمود و اثر این دو با هم قویتر از اثر آنها به تنهایی بود. هیچ کدام از عصاره ها روی فشار خون طبیعی تاثیری نداشتند. نتیجه گیری : بنا به نتایج بدست آمده مصرف مقادیر مشخصی از برگ زیتون و برگ شاه توت خواص هیپوکلسترولمیک و آنتی آترواسکلروتیک دارد اما هیچ گونه اثری روی فشار خون طبیعی ندارد.

Title: صدمات تراکئو برونشیال در ترومای غیر نافذ قفسه سینه

http://yafte.lums.ac.ir/article-1-956-fa.html

مقدمه : صدمات تراکئو برونشیال عارضه نادر و گاها“ کشنده ای است که در موارد معدودی از ترومای غیر نافذ قفسه سینه دیده می شود و اگر زود تشخیص داده نشود, عوارض و مرگ و میر بالائی خواهد داشت. آمار 7 ساله این موارد از 4 مرکز تروما در لس آنجلس 9 مورد بوده است؛ ولی اخیرا“ در مقالات علمی موارد بیشتری از این تروما گزارش شده و علت این افزایش را وجود مراکز پیشرفته و تشخیص زودرس دانسته اند . پارگی برونش راست شایع تر است و اغلب این موارد با شکستگی دنده و ترقوه همراه هستند. در این مطالعه صدمات تراکئوبرونشیال در ترومای غیر نافذ قفسه سینه هدف بررسی بوده است. گزارش موارد: در این مقاله اطلاعات بالینی و پاراکلینیک 3 بیمار که به علت صدمات غیر نافذ تراکئوبرونشیال در 10 سال اخیر به ما مراجعه کرده اند از جمله تظاهرات بالینی و یافته های تشخیصی , نوع درمان و سیر بالینی مورد بررسی قرار گرفته است. در هر 3 بیمار , که به فاصله 5-2 ساعت از بروز حادثه تشخیص داده شده و تحت عمل جراحی قرار گرفته اند نتیجه رضایت بخش بوده و مرگ و میر نداشته ایم . نتیجه گیری: این نتایج با دستاوردهای مطالعات دیگر که بر اهمیت تشخیص و درمان زود هنگام این گونه صدمات تاکید کرده اند هماهنگ است.

Title: ارتباط بین هلیکوباکترپیلوری و استفراغ های شدید حاملگی در زنان باردار شهرستان خرم آباد ( سال 81-1380)

http://yafte.lums.ac.ir/article-1-957-fa.html

مقدمه : استفراغ یکی از شایع ترین مشکلات دوران بارداری است که در 50% حاملگی ها اتفاق می افتد.استفراغ های شدید حاملگی وضعیت نادری است که تقریبا 1 در500 حاملگی ها اتفاق می افتد . اگر چه علت استفراغ های شدید نامشخص است؛ ولی چندین مطالعه ارتباط هلیکوباکترپیلوری را با بروز این مشکل مطرح می نماید . با توجه به این امر, پژوهش حاضر با هدف بررسی ارتباط هلیکو باکترپیلوری و استفراغ های شدید حاملگی و مقایسه آن با افراد سالم انجام شد . مواد و روشها : این پژوهش یک بررسی مورد ـ شاهدی است که درآن 200 نفر از زنان باردار به عنوان گروه شاهد و مورد در درمانگاه های شهرستان خرم آباد مورد بررسی قرار گرفتند . تمامی افراد باردار و از نظر تعدادجنین تک قلو بودند. از افراد واجد شرایط نمونه گیری انجام شد وبا استفاده از آزمایشات سرولوژیک و روش ELISA غلظتIgG درافراد مورد و شاهد مورد ارزیابی قرار گرفت . یافته ها : نتایج بدست آمده نشان داد, شیوع عفونت هلیکوباکترپیلوری در گروه مورد8/79% نسبت به گروه کنترل (8/46%) با001/0 p< اختلاف معنی داری را نشان می دهد . همچنین بین دو گروه از نظر سن , سن حاملگی , وضعیت اقتصادی - اجتماعی و سطح تحصیلات تفاوت معنی داری وجود نداشت . نتیجه گیری : نتایج مطالعه نشان می دهد هلیکوباکترپیلوری نقش مهمی در اتیولوژی استفراغ های شدید حاملگی به عهده دارد .

Title: مقایسه لوله گذاری تراشه با دو روش بلوک عصبی و تزریق وریدی

http://yafte.lums.ac.ir/article-1-958-fa.html

مقدمه : از آنجا که اولین اقدام در برخورد با بیماران بدحال , نگهداری راه هوائی است و هر گونه تعلل در اینکار عواقب خطرناکی ازجمله آسپیراسیون و در نهایت مرگ را بدنبال دارد ، لذا بسیاری از این بیماران نیازمند لوله گذاری تراشه می باشند. روش متداول لوله گذاری تراشه ،تزریق وریدی دیازپام و پتیدین و گاهی شل کننده های عضلانی می باشد ؛ ولی دربیشتر بیماران با ≤7 GCS, استفاده از داروهای مذکور سبب کاهش بیشترسطح هوشیاری می گردد. هدف از انجام این مطالعه مقایسه لوله گذاری تراشه با دو روش بلوک عصبی و تزریق وریدی بود. مواد و روشها : در این کار آزمایی بالینی 60 بیمار نیازمند لوله گذاری تراشه, به صورت تصادفی در دو گروه 30 نفره تقسیم شدند. قبل از لوله گذاری در گروه اول تزریق وریدی پتیدین ،لیدوکائین و دیازپام انجام شد و در گروه دوم با استفاده از لیدوکائین2 % عصب سوپرالارنجیال دو طرفه و ترا نس تراکئال بلوک شدند .هر دو گروه بیماران ازنظر عوارض لوله گذاری مانندشلی فک، حرکت تارهای صوتی و زورزدن مورد مقایسه قرار گرفتند . یافته ها : آزمون آماری نشان داد در گروه بلوک عصبی در مقایسه با گروه تزریق وریدی حرکت تارهای صوتی ، قدرت زورزدن بمراتب کمتر و شلی فک بیشتر بود ( 001/0 p<) . نتیجه گیری: براساس نتایج بدست آمده استفاده از بلوک عصبی دربیماران با ≤7 GCS بیماران پیشنهاد می شود.

Title: درمان غیر جراحی انتخابی در اصابت گلوله به شکم

http://yafte.lums.ac.ir/article-1-959-fa.html

مقدمه : اثرات ناشی از اصابت گلوله و ترکشهای با سرعت بالا (High velocity bullets ) به خصوص اثرات موج انفجاری آنها روی احشاء داخل شکم امری بدیهی است. اکثر جراحان عقیده دارند که در صورت اصابت گلوله به جدار شکم, بیمار بایستی تحت عمل لاپاراتومی تجسمی قرار گیرد تا ضایعات مخفی نیز کشف و درمان شود . با توجه به وجود موارد زیادی لاپاراتومی منفی و پیشرفت تکنیکهای رادیولوژیک (سونوگرافی , سی تی اسکن و MRI ) پیشنهاد شده است که این بیماران در صورت نداشتن علائم بالینی دال بر صدمات احشایی داخل شکمی به روش غیر جراحی انتخابی درمان شوند. هدف این مطالعه بررسی درمانهای غیر جراحی در اصابت گلوله به شکم بود. مواد و روشها : مطالعه به صورت مقاله مروری است و گزارشات منتشر شده از طریق اطلاعات موجود در مدلاین و کتب مرجع جراحی و تروما بررسی گردید و مباحث مربوط به معایب و محاسن این روش درمانی تجزیه و تحلیل شد. یافته ها و نتیجه گیری : با گرایش روز افزون پزشکی به روش های غیر جراحی و غیر تهاجمی, امروزه بیمارانی که ترومای گلوله به جدار شکم دارند, می توانند درمان حمایتی شوند و نتایج رضایت بخشی نیز داشته باشند . مطالعه مروری ما نشان داده است که درمان انتخابی غیر جراحی در بیمارانی که گلوله به شکم آنها اصابت کرده, یک روش مناسب و مطمئنی است, و لاپاراتومی های غیر ضروری و هزینه های بیمارستانی را بطور چشمگیری کاهش می دهد در این روش درمانی بایستی احتیاط لازم به عمل آید تا سلامت این روش درمانی در مطالعات کار آزمایی بالینی بعدی ثابت شود.

Title: عوارض پوستی آمپول Lipiodol در شهر خرم آباد

http://yafte.lums.ac.ir/article-1-960-fa.html

مقدمه : عوارض ناشی از کمبود ید گریبان گیر میلیونها نفر در سراسر جهان است . نمک ید دار و ید محلول در روغن برای جبران این کمبود مناسب است؛ بطوریکه یک دوز از ید محلول در روغن می تواند کمبود ید را برای 5-3 سال تصحیح نماید. هدف از انجام این مطالعه بررسی عوارض پوستی آمپول لیپیدول در شهر خرم آباد بود. مواد و روشها : در این مطالعه به بررسی عوارض پوستی ناشی از تزریق ید به میزان 1-5/0 میلی لیتر از ید محلول در روغن Lipiodol در 116 بیمار که با عوارض پوستی به درمانگاه تخصصی پوست شهر خرم آباد طی نیمه اول سال 76 مراجعه کرده بودند, مورد بررسی قرار گرفت. ابزار گردآوری اطلاعات پرسشنامه بود. یافته ها : از 116 نفر مراجعه کننده 115 نفر زن و 1 نفر مرد بودند . حدود 78% عوارض پوستی در عرض یکماه پس از تزریق روی داده بود. از نظر سنی نیز بیشتر عوارض در گروه سنی 33-24 سال (96/43%) قرار داشتند . 8/25% دارای سابقه حساسیت به انواع داروها و غذاها بودند. اکثر ضایعات 3/91% محدود به ناحیه تزریق بوده (بازو یا باسن) و در بقیه موارد هم (7/8%) به صورت ژنرالیزه بوده است . ضایعات عمدتا“ با خارش در ناحیه تزریق شروع شده؛ سپس یکسری راشهای اریتماتو با ادم موضعی نیز به آن اضافه شده بود. در تعدادی از موارد, ادم و اریتم کل اندام را گرفتار کرده بود. نتیجه گیری : بنا به اطلاعات موجود می توان استنباط کرد که مکانیزم احتمال بروز ضایعات پوستی افزایش واکنش حساسیتی تاخیری می باشد (Delayed Hypersensivity) . بنابراین با توجه به بروز بسیار کم عوارض پوستی ناشی از تزریق Lipiodol , هنوز هم این روش ید رسانی در مناطق هیپراندمیک گواتر می تواند مفید باشد؛البته مشروط به اینکه برای افراد پر خطر تمهیدات ویژه در نظر گرفته شود.

Title: بررسی 212 مورد التهاب مزمن گوش میانی در بیماران ارجاعی به بیمارستان امیر اعلم تهران در شش ماه اول سال 1380

http://yafte.lums.ac.ir/article-1-961-fa.html

مقدمه : التهاب گوش میانی یکی از عفونتهای شایع کودکان است. بالغ بر 80% کودکان سنین یک تا شش سال به عفونت حاد باکتریال گوش میانی دچار می شوند و علی رغم استفاده وسیع آنتی بیوتیک ها در درمان التهاب گوش میانی عوارض عفونی به وفور در این بیماران دیده می شود. هدف از انجام این مطالعه بررسی التهاب مزمن گوش بود. مواد و روشها : روش این مطالعه توصیفی است که با استفاده از پرونده بیماران به بررسی مشخصات کلینیکی ، نحوه درمان و اتیولوژی کلیه بیمارانی که طی شش ماه اول سال 1380 , التهاب مزمن گوش میانی به بیمارستان امیر اعلم تهران مراجعه کرده بودند , پرداخته شد. یافته ها : نتایج مطالعه نشان داد که 212 بیمار جهت درمان در طول شش ماهه اول سال به بیمارستان مراجعه نمودند . در بیشتر بیماران ،‌بیماری در سنین بلوغ پیدا شده بود. نسبت مرد به زن مساوی بود . شکایت عمده بیماران درهنگام مراجعه در 115 مورد (54%) ترشح از گوش و 55 مورد (26%) کاهش شنوایی و بقیه شامل سردرد ، تهوع،‌استفراغ،‌تب ولرز بود.یافته های پاتوبیولوژیک نشان داد که 120 مورد (57%) دارای پارگی بزرگ مرکزی ، 82 مورد (39%) کلستاتوم،80 مورد(38%) پارگی حاشیه ای خلفی فوقانی و بقیه شامل تمپانو اسکلروز ، پولیپ ، بافت گرانولوسیون ،اوتیت میانی و چسبنده و کلستاتوم مادر زادی بود . گزارش اتاق عمل نشان میدهد که در 76% بیماران تخریب زنجیره استخوانی وجود داشت . نتیجه گیری : نتایج این مطالعه نشان داد که بیماری التهاب مزمن گوش میانی در ایران بیشتر در سنین بالا بوده که میتواند به علت سابقه بیماری در کودکی و عدم درمان به موقع و کامل بیماری در سنین کودکی باشد که این خود میتواند موضوع مطالعات بیشتر در این زمینه باشد .

Title: شناسایی و تعیین فون عقربهای شهرستان خرم آباد

http://yafte.lums.ac.ir/article-1-962-fa.html

مقدمه : مطالعات مربوط به بیولوژی و اکولوژی عقربها در ایران با محدودیت شدیدی مواجهه است و این مطالعات می بایست با شناسایی زیستگاهها و فون عقربهای هر منطقه در کشور آغاز و با بررسی خصوصیات زیستی (بیولوژی ) و زیست محیطی (اکولوژی ) آنها کامل گردد. هدف از این بررسی نیز شناسایی و تعیین فون عقربهای استان لرستان بود. مواد و روشها : در این مطالعه توصیفی پنج منطقه از شهرستان خرم آباد تاشعاع بیست کیلومتری از هر طرف شهر انتخاب و در دو فصل بهار و تابستان با تعیین دمای محیط اقدام به صید عقرب گردید . در مجموع 52 عقرب از این مناطق صید شد.جهت تغذیه عقربها از بندپایان مختلف مثل سوسرهای شرقی Blatta orientalis و آلمانی Blatella gremania لارو پروانه , خرخاکی و مگس بالغ خانگی در آزمایشگاه استفاده شد تا تمایل تغذیه ای عقرب ها مشخص شود. یافته ها: بر اساس نتایج بدست آمده, از مجموع 52 عقرب صید شده 29 عقرب آن از خانواده بوتیدهButhidae و 23 عقرب از خانواده اسکورپیونیده Scorpionidae بودند . وفور جمعییت برای خانواده بوتیده در شهرستان خرم آباد 56% و برای خانواده اسکورپیونیده 44% می باشد که برای هر دو خانواده با توجه به مدت زمان محدود نمونه برداری وفور قابل توجه ونسبتا بالایی را نشان می دهد . برای نگهداری عقربها در آزمایشگاه لانه های مصنوعی از مقوا تهیه و در دسترس آنها قرار داده شد که به خوبی مورد استقبال واقع گردید. بیشترین تمایل تغذیه ای عقرب به مگس خانگی بالغ بود. نتیجه گیری : بر اساس نتایج بدست آمده بیشتر عقربها مربوط به خانواده بوتیده و از نوع Buthuthus Snulcyi بود که این عقرب دارای نیش دردناکی است و روی سیستم عصبی تاثیر می گذارد .در کودکان و افراد مسن بسیار کشنده است. لذا شناخت این گونه راههای مقابله با آن از اهمیت زیادی برخوردار است.

Title: ضایعات پوستی در جانبازان شیمیایی استان لرستان

http://yafte.lums.ac.ir/article-1-965-fa.html

مقدمه : سلاحهای شیمیایی که سابقه آنها به جنگ جهانی اول بر می گردد به کلیه عواملی اطلاق می شود که اثرات مستقیم سمی بر روی انسان , حیوانات و گیاهان دارند. رژیم عراق در طی دوران جنگ تحمیلی از سموم مختلف به خصوص گاز خردل و اعصاب به طور قابل ملاحظه ای استفاده نمود که اثرات آن پس از گذشت سالها همچنان مشهود است. مواد و روشها : این مطالعه توصیفی ـ مقطعی با هدف بررسی ضایعات پوستی در جانبازان شیمیایی استان لرستان در سال 81 انجام شد. اطلاعات به وسیله معاینه بالینی جمع آوری و در پرسشنامه ثبت شد و در مجموع 95جانباز که در دوران جنگ در معرض مستقیم با گازهای شیمیایی بودند, مورد بررسی قرار گرفتند . یافته ها :‌کلیه جانبازان شیمیایی مورد مطالعه , مرد با میانگین سنی 26/39سال و همگی مبتلا به ضایعات پوستی بودند. شایعترین علائم پوستی: خارش ، سوزش ، خشکی و پوسته پوسته شدن بود.از نظر نوع ضایعه, اریتم (81%)، اکسکوریاسیون (9/87%)و پاپول (5/49%)فراوانترین ضایعات پوستی بودند. تشخیص نهایی در 78درصد جانبازان شیمیایی مورد مطالعه درماتیت مزمن و 7/7درصد درماتیت سبوره ئیک و 8/8درصد درماتیت مزمن و سبوره ئیک به صورت توأم بوده است .در هنگام مواجهه با گازهای شیمیایی فقط 9/37درصد جانبازان از ماسک و 40درصد از لباس بطور کامل و صحیح استفاده کرده و بقیه یا استفاده نکرده یا بطور ناقص این وسایل را به کاربرده بودند . ضایعات اکثراًدر نواحی سینه ، کمر و پشت ، اندام تحتانی ، شکم و سرو صورت بوده و 78درصد جانبازان دارای ضایعات متعدد بودند. نتیجه گیری : با توجه به نتایج این مطالعه, کلیه جانبازان شیمیایی این استان از ضایعات پوستی مختلف و با درجات متفاوت رنج می برند. هر چند پیش از نیمی از آنان نوع گاز را نمی دانستند . لذا پیشنهاد می شود مطالعات گسترده تری بر روی اثرات دراز مدت این گازها انجام شود.

Title: نگرش دانش آموزان دبیرستانی شهر خرم آباد نسبت به اختلالهای روانی

http://yafte.lums.ac.ir/article-1-966-fa.html

مقدمه : در سالهای اخیر مطالعات گوناگونی در رابطه با نگرش اقشار مختلف جامعه نسبت به بیماری های روانی انجام شده است که نتایج آن توانسته است با برنامه ریزی بهینه در این مقوله، تحولات مثبتی در بهبود نگرش جامعه ایجاد نماید. هدف از انجام این مطالعه نیز بررسی نگرش دانش آموزان دبیرستانی نسبت به اختلالهای روانی بود. مواد و روشها : در این مطالعه توصیفی - مقطعی به بررسی نگرش دانش آموزان دبیرستانی نسبت به اختلالهای روانی پرداخته شد. تعداد 600 دانش آموز (شامل 301 دانش آموز دختر و 299 دانش آموز پسر)به صورت تصادفی چند مرحله ای انتخاب شدند. ابزار گرد آوری اطلاعات یک پرسشنامه شامل: سوالاتی پیرامون مشخصات دموگرافیک و سنجش نگرش دانش آموزان بود. اطلاعات به دست آمده توسط نرم افزار9.1 SPSS تجزیه و تحلیل شدند. یافته ها : نتایج بدست آمده نشان داد که 8/58 درصد دانش آموزان دارای نگرش منفی و 2/41 درصد دارای نگرش مثبت نسبت به اختلالهای اعصاب و روان بودند.آزمون آماری t اختلاف معنی داری بین میزان نوع نگرش دانش آموزان دختر و پسر نشان نداد(05/0 (p> . بین پایه تحصیلی با نگرش دانش آموزان نسبت به اختلالات اعصاب و روان از لحاظ آماری رابطه معنی دار وجودداشت (05/0p<). 77 درصد دانش آموزان اظهار داشتند در صورت برخورد با بیماران مبتلا به اختلال اعصاب و روان به آنها توصیه مراجعه به پزشک و افراد واجد صلاحیت را می دهند. 54 درصد دانش آموزان، عدم آگاهی و توجه والدین به فرزندانشان را در بروز اختلالهای اعصاب و روان مؤثر دانستند. 3/46 درصد تنبیه بدنی از طرف معلم و مسئولین مدرسه را در بروز اختلالات اعصاب و روان مؤثر دانستند. 90% آنها به آموزش از طریق روانپزشک یا روانشناس در مدرسه علاقه زیادی نشان دادند. نتیجه گیری : نتایج این مطالعه نشان می دهد نگرش دانش آموزان دبیرستانی شهر خرم آباد نسبت به اختلالات اعصاب و روان در سطح مطلوبی نیست. با توجه به اینکه ریشه بیشتر اختلالهای اعصاب و روان از عدم آگاهی و نگرش نادرست به این مقوله است، ضروری است که با نگاهی ژرف به این مسئله با برنامه ریزی صحیح در جهت تغییر نگرش دانش آموزان گام مؤثری در جهت سلامت و بهداشت روانی نوجوانان برداشته شود.

Title: ویژگیهای معتادین خود معرف شهرستان الیگودرز

http://yafte.lums.ac.ir/article-1-967-fa.html

مقدمه : کیست مزانتریک, توده های داخل شکمی نادری هستند که شیوع آنها در افراد بالغ یک در 140000 مورد در بیمارستانهای جنرال گزارش شده است . این بیماری بیشتر در کودکان مشاهده می شود و شیوع آنها یک مورد در 20000 در بیمارستانهای اطفال است. کیست‌های مزانتر اکثرا“ دارای ماهیت خوش خیم بوده و با تظاهرات بالینی بسیار متنوعی باعث مراجعه به پزشک می شوند . تشخیص بالینی آنها بسیار مشکل است. و ظن قوی به کیست با کمک گرفتن از روش های پاراکلینیک تشخیص را مسجل می کند. در این مطالعه یک مورد کیست مزانتر با تظاهرات غیر معمول مورد بررسی قرار گرفته است. معرفی بیمار : بیمار معرفی شده آقای 50 ساله ‌ای است که با شکایت از اتساع شکم به همراه درد مبهم مراجعه کرده بود. در معاینه شکم ,توده‌ای واضح لمس نشد؛ اما دیستانسیون منتشر وجود داشت . برای بیمار در ابتدا تشخیص انسداد روده مطرح بود؛ اما با توجه به یافته های تصویر برداری ، سونوگرافی ونمای سی تی اسکن و در پی شک بالینی ,بیمار با تشخیص کسیت مزانتر مورد عمل جراحی قرار گرفت و کیست‌های متعددی از شکم بیمار خارج شد. پاتولوژی توده ها Lymphatic cysts of mesentry گزارش گردید . بیمار با حال عمومی خوب مرخص شد.

Title: گزارش یک مورد کیست مزانتر با تظاهرات غیر معمول

http://yafte.lums.ac.ir/article-1-968-fa.html

مقدمه : کیست مزانتریک, توده های داخل شکمی نادری هستند که شیوع آنها در افراد بالغ یک در 140000 مورد در بیمارستانهای جنرال گزارش شده است . این بیماری بیشتر در کودکان مشاهده می شود و شیوع آنها یک مورد در 20000 در بیمارستانهای اطفال است. کیست‌های مزانتر اکثرا“ دارای ماهیت خوش خیم بوده و با تظاهرات بالینی بسیار متنوعی باعث مراجعه به پزشک می شوند . تشخیص بالینی آنها بسیار مشکل است. و ظن قوی به کیست با کمک گرفتن از روش های پاراکلینیک تشخیص را مسجل می کند. در این مطالعه یک مورد کیست مزانتر با تظاهرات غیر معمول مورد بررسی قرار گرفته است. معرفی بیمار : بیمار معرفی شده آقای 50 ساله ‌ای است که با شکایت از اتساع شکم به همراه درد مبهم مراجعه کرده بود. در معاینه شکم ,توده‌ای واضح لمس نشد؛ اما دیستانسیون منتشر وجود داشت . برای بیمار در ابتدا تشخیص انسداد روده مطرح بود؛ اما با توجه به یافته های تصویر برداری ، سونوگرافی ونمای سی تی اسکن و در پی شک بالینی ,بیمار با تشخیص کسیت مزانتر مورد عمل جراحی قرار گرفت و کیست‌های متعددی از شکم بیمار خارج شد. پاتولوژی توده ها Lymphatic cysts of mesentry گزارش گردید . بیمار با حال عمومی خوب مرخص شد.

Title: گزارش یک مورد بارداری چهار قلو خود به خودی نادر در اصفهان

http://yafte.lums.ac.ir/article-1-969-fa.html

بارداری چهار قلو خود به خودی یک رخداد نادر است. که با عوارض شدید سقط , زایمان زودرس , خونریزی سه ماهه اول و کم خونی , .... همراه است. ‌بیمار مورد معرفی خانمی 29 ساله با سابقه یک مورد بارداری دوقلوی نافرجام است .در ماه سوم بارداری دوم, با انجام سونوگرافی متوجه چهار قلو بودن جنین می شود که عمل سرکلاژ برای وی انجام می گیرد. در 26 هفتگی با تشخیص زایمان زود رس در بیمارستان الزهراء بستری می شود و در سن 29 هفته و دو روز بارداری, تحت سزارین قرار می گیرد . از چهار نوزاد متولد شده دو نوزاد دختر و یک نوزاد پسر سالم هستند و نوزاد پسر دیگر به علت سندرم زجر تنفسی بعد از 24 ساعت فوت کرد.
